# Leaf Shedding and Non-Stomatal Limitations of Photosynthesis Mitigate Hydraulic Conductance Losses in Scots Pine Saplings During Severe Drought Stress

**DOI:** 10.3389/fpls.2021.715127

**Published:** 2021-09-03

**Authors:** Daniel Nadal-Sala, Rüdiger Grote, Benjamin Birami, Timo Knüver, Romy Rehschuh, Selina Schwarz, Nadine K. Ruehr

**Affiliations:** ^1^Karlsruhe Institute of Technology, Institute of Meteorology and Climate Research - Atmospheric Environmental Research (IMK-IFU), Garmisch-Partenkirchen, Germany; ^2^University of Bayreuth, Chair of Plant Ecology, Bayreuth, Germany; ^3^Department of Botany, University of Innsbruck, Innsbruck, Austria

**Keywords:** leaf shedding, non-stomatal limitations of photosynthesis, Scots pine, tree hydraulic simulation models, xylem vulnerability

## Abstract

During drought, trees reduce water loss and hydraulic failure by closing their stomata, which also limits photosynthesis. Under severe drought stress, other acclimation mechanisms are trigged to further reduce transpiration to prevent irreversible conductance loss. Here, we investigate two of them: the reversible impacts on the photosynthetic apparatus, lumped as non-stomatal limitations (NSL) of photosynthesis, and the irreversible effect of premature leaf shedding. We integrate NSL and leaf shedding with a state-of-the-art tree hydraulic simulation model (SOX+) and parameterize them with example field measurements to demonstrate the stress-mitigating impact of these processes. We measured xylem vulnerability, transpiration, and leaf litter fall dynamics in *Pinus sylvestris* (L.) saplings grown for 54 days under severe dry-down. The observations showed that, once transpiration stopped, the rate of leaf shedding strongly increased until about 30% of leaf area was lost on average. We trained the SOX+ model with the observations and simulated changes in root-to-canopy conductance with and without including NSL and leaf shedding. Accounting for NSL improved model representation of transpiration, while model projections about root-to-canopy conductance loss were reduced by an overall 6%. Together, NSL and observed leaf shedding reduced projected losses in conductance by about 13%. In summary, the results highlight the importance of other than purely stomatal conductance-driven adjustments of drought resistance in Scots pine. Accounting for acclimation responses to drought, such as morphological (leaf shedding) and physiological (NSL) adjustments, has the potential to improve tree hydraulic simulation models, particularly when applied in predicting drought-induced tree mortality.

## Introduction

There is increasing evidence that hydraulic failure is a main trigger of tree death in response to drought and hot drought (Allen et al., 2015; McDowell et al., 2018; Brodribb et al., 2020). While trees are well-adapted to respond to seasonal and short-term increases in soil and atmospheric drought, extreme climatic conditions, e.g., anomalously high summer temperatures coupled with low soil water availability, as experienced, for instance, in central Europe during the summer of 2018 (Hari et al., 2020; Schuldt et al., 2020), can cause substantial hot drought-induced damage. Increasing soil and atmospheric drought result in increasing tree internal water column tensions. As such tensions rise, air bubbles, i.e., emboli, can form in the xylem, reducing xylem hydraulic conductance (Tyree and Ewers, 1991). Reduced xylem conductance limits water transport upward, from soil to the leaves, which may lead to dehydration of cambium and apical meristems, canopy dieback, and ultimately tree death (e.g., Carnicer et al., 2011; Anderegg et al., 2012; Allen et al., 2015; Choat et al., 2018; Reich et al., 2018; Hesse et al., 2019).

It is well-established that stomatal closure is the first and foremost mechanism that limits water loss and buildup of excessive xylem tension (Hall and Kaufmann, 1975; Monteith, 1995; Choat et al., 2018). This comes at a cost of reduced leaf permeability to CO_2_, which limits C assimilation (Martorell et al., 2014; Reich et al., 2018). Much research has focused on modeling stomatal conductance (gs) to water deficit (Damour et al., 2010; Mencuccini et al., 2019), as well as tree internal water balance after stomatal closure (e.g., Martin-St. Paul et al., 2017; Cochard et al., 2021). To date, many approaches exist that combine gs responses to decreasing soil water content (SWC) and increasing vapor pressure deficit (VPD). The approaches range from empirical relationships (e.g., Leuning, 1995), to mechanistical descriptions based on optimality theory such as maximizing C gain per unit of transpiration (e.g., Medlyn et al., 2011), maximizing transpiration while reducing conductivity loss (Sperry and Love, 2015), or maximizing C gain while minimizing loss in hydraulic conductivity (e.g., Sperry et al., 2017; Eller et al., 2018; 2020). The success of these models has been mixed, leading to a good representation of broad monthly and annual transpiration and productivity patterns but often failing to capture subtler responses arising when drought stress intensifies (e.g., Drake et al., 2017; Yang et al., 2019; De Kauwe et al., 2020; Bassiouni and Vico, 2021; Mu et al., 2021; Nadal-Sala et al., 2021). Hence, challenges to model tree drought responses and mortality persists albeit increasing developments of optimization-based tree hydraulic models over the recent years. Therefore, further model improvements regarding tree acclimation responses to drought beyond stomatal closure have been recommended (e.g., Keenan et al., 2010; Wolfe et al., 2016; Martin-St. Paul et al., 2017; Sperry et al., 2019; Gourlez de la Motte et al., 2020). To do so, controlled experiments that address specific tree physiological responses to drought provide an opportunity to improve and evaluate the performance of tree hydraulic models (e.g., Hartig et al., 2012; Medlyn et al., 2015; Dietze et al., 2018).

Under sustained drought, stomatal regulation in response to CO_2_ demand on the one hand and evaporation demand on the other may not be enough to mitigate hydraulic tension and prevent embolism formation in the xylem. Other responses are, thus, often triggered to reduce water loss, such as metabolic changes or increased internal resistance that then feedback to stomatal conductance, as well as accelerated senescence of various tissues. In particular, many studies report a slowdown of the photosynthetic activity during drought (e.g., Xu and Baldocchi, 2003; Keenan et al., 2010; Yang et al., 2019; Gourlez de la Motte et al., 2020). Such slowdown may have different causes such as increased mesophyll resistance (Flexas et al., 2007, 2012; Evans, 2021), drought-related enzymatic down-regulation (e.g., Flexas et al., 2004; Niinemets and Sack, 2006; Niinemets et al., 2006; Sugiura et al., 2020), and/or decreasing carbon demand (Fatichi et al., 2014). Since all these mechanisms lead to a reduction in water loss, here, we consider them in a lumped manner as non-stomatal limitations of photosynthesis (NSL), thereby enabling the consideration of this additional response process in models that are simulating stand productivity and transpiration in dependence on water availability (Zhou et al., 2013; Drake et al., 2017; Yang et al., 2019).

Another key mechanism of how trees can respond to drought is reducing their leaf area (e.g., Munné-Bosch and Alegre, 2004; Sala et al., 2010; Martin-St. Paul et al., 2013; Wolfe et al., 2016; Hochberg et al., 2017; Li et al., 2020; Schuldt et al., 2020). Drought-induced leaf senescence reduces total tree transpiration, at the expense of growth at mid-term, as rebuilding canopy structure requires extra C investment, either from non-structural carbohydrate reserves or from the assimilation of the remaining or newly grown leaves once drought stress has been released (Yan et al., 2017; Ruehr et al., 2019). Additionally, shedding leaves without full nutrient resorption imply net nutrient losses (Marchin et al., 2010; Chen et al., 2015), which may further limit photosynthesis post-drought with consequences for tree performance in the long term. Leaf shedding tends to occur after stomata closure; hence, it mainly reduces marginal loss in water *via* residual cuticular conductance and incomplete stomatal closure (e.g., Martin-St. Paul et al., 2017; Cardoso et al., 2020; Li et al., 2020). Under sustained drought, such water loss may be critical for tree survival (e.g., Blackman et al., 2016, 2019), especially considering that residual cuticular conductance increases with temperature (Schuster et al., 2016), leading to faster dehydration of plants particularly during heat waves. While leaf shedding, in response to drought, is routine in drought-deciduous trees (e.g., Ichie et al., 2004; Pineda-García et al., 2013; Ruehr et al., 2016), it can be rather seen as an emergency response in temperate conifers, with profound consequences for drought recovery.

Here, we aim to quantify the importance of NSL and leaf shedding mechanisms regarding hydraulic safety. To do so, we measured hydraulic vulnerability, and transpiration and leaf shedding dynamics in potted *P. sylvestris* L. saplings exposed to 2-month severe dry-down. Then, we trained a big-leaf canopy gas exchange simulation model based on the optimization of stomatal conductance as xylem tension increases (SOX model, Eller et al., 2018, 2020). The SOX model assumes that trees regulate stomatal conductance to maximize C uptake while minimizing loss in soil-to-root hydraulic conductance. Once the model was trained, we evaluated the importance of NSL and leaf shedding for hydraulic regulation. The initial hypotheses were the following: (1) including non-stomatal limitations of photosynthesis will improve the representation of transpiration during drought; (2) seasonal leaf shedding in pine trees is coordinated with stomata closure to reduce dehydration, and (3) according to the “leaf fuse” hypothesis (e.g., Hochberg et al., 2017) leaf shedding will mitigate mid-term losses in hydraulic conductance.

## Materials and Methods

### Experimental Setup

Potted *P sylvestris* L. saplings were grown in Garmisch-Partenkirchen, Germany (708 m above sea level, 47°28′32.9″N, 11°3′44.2″E). Three-year-old Scots pine saplings were purchased from a local tree nursery in 2018 and planted in individual pots (120 l, 55 cm in diameter, 70 cm in height; Brute, Rubbermaid, Atlanta, GA, United States) in a 6:3:1 mixture of potting substrate (No. 170, Klasmann-Deilmann, Geeste, Germany), perlite (Perligran Premium, Knauf Performance Materials GmbH, Dortmund, Germany), and quartz sand (3–6 and 0.1–0.3 mm). Slow-release fertilizer (100 g, Osmocote® Exact Standard 5-6M 15-9-12+2MgO+TE, ICL Specialty Fertilizers Benelux, The Netherlands) was added to the mixture and supplemented by liquid fertilizer (Manna® Wuxal Super; Wilhelm HaugGmbh, Ammerbuch, Germany). From May to October 2019, the saplings were kept inside the adjacent greenhouse and exposed to mild soil water limitation with air temperature ranging between 10 and 35°C, to prime the trees for the upcoming experiment. From October 2019 to July 2020, the saplings were again grown outside and irrigated once a week from May 2020 onwards. After leaf elongation was finished (mid of July), the then 5-year-old saplings (*n* = 16) were transferred to the greenhouse once more and irrigated to field capacity (SWC ~0.35 m^3^ m^−3^) before the drought experiment was started. To minimize soil evaporation, the top of the soil was covered with an opaque plastic sheet, which was periodically ventilated.

**Figure 1:**
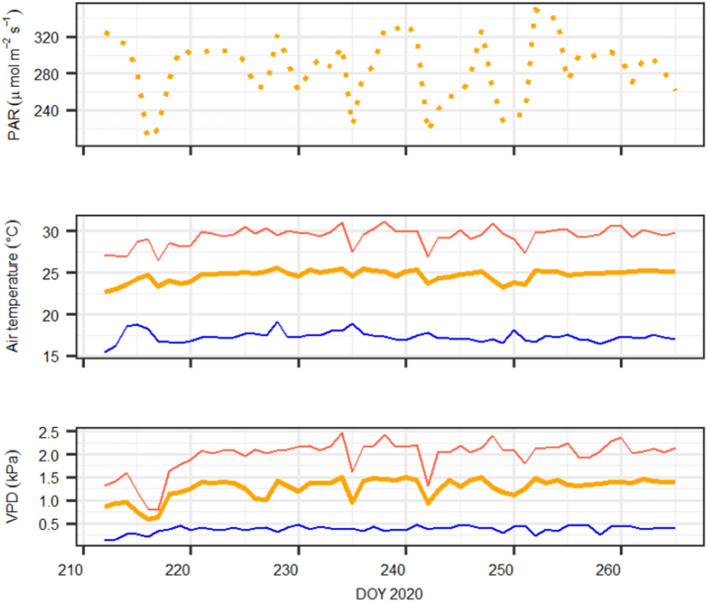
Daily meteorological conditions during the experiment. Upper panel: daylight average photosynthetic active radiation (PAR, in μmol m^−2^ s^−1^); middle panel: maximum (red), minimum (blue), and average (orange) air temperature (in °C); bottom panel: maximum (red), minimum (blue), and average (orange) atmospheric vapor pressure deficit (VPD, in kPa). Note that on DOY 216–217, vapor pressure deficit (VPD) and temperature conditions were lowest because of a cold weather front that was not compensated by heating the greenhouse.

The greenhouse is equipped with special UV-transmissive glass, and incoming light was supplemented with plant growth lamps (T-agro 400 W; Philips, Hamburg, Germany). Air temperature and air humidity were computer-regulated (CC600, RAM Regel- und Messtechnische Apparate GmbH, Herrsching, Germany). Environmental conditions at canopy height such as photosynthetic active radiation (PQS 1, Kipp&Zonen, Delft, The Netherlands), air temperature, and relative humidity (CS215, Campbell Scientific Inc., Logan, UT, United States) were monitored and logged at 10-min intervals (CR1000; Campbell Scientific Inc., Logan, UT, United States). The environmental conditions during the experiment are shown in Figure 1.

### Soil Water Content and Transpiration

We continuously measured soil water content (SWC) and sap flow in six randomly selected saplings. Measurements started on DOY 206, but the first week was excluded from this analysis because of plant acclimation to the new conditions, and sensor malfunction and re-calibration (data not shown). Therefore, valid measurements started on DOY 212. Volumetric SWC was monitored at 0–10 c and 40–50 cm depth (10HS; Decagon Devices Inc., Pullman, WA, United States, Supplementary Figure 1) to cover the entire rooting area. To estimate the water available for each tree, SWC data from the two depths was averaged. Each SWC sensor was pre-calibrated to the potting medium following the recommendation of the manufacturer. Stem sap flow was measured using the heat-balance method (EMS 62, EMS, Brno, Czech Republic). Sap flow sensors were installed in the upper part of the canopy (height ~1–1.5 m) as the cylindrical build does not support stem diameters >2 cm. The sensors were shielded with aluminum bubble foil to minimize error due to temperature fluctuations. Sap flow measurements were stored in 30-min intervals. Daily whole-tree transpiration was calculated by assuming that transpiration per unit of leaf area (see section Tree Biomass Compartment Measurement) was equal above and below the sensor, as in a homogeneously coupled non-shadowed canopy we expected the environmental drivers to be homogeneous for the leaves above and below. Therefore, transpiration was calculated as the sum of the measured sap flow plus the sap flow needed to supply the transpiration of the leaves below the sensor (Equation 1)

(1) $$\begin{array}{l} Tr=J*\left(1+\frac{LA_{below}}{LA_{above}}\;\right) \end{array}$$

where Tr is the daily whole-tree transpiration (in kg day^−1^ tree^−1^), J is the daily sap flow (in kg day^−1^ tree^−1^), and LA_below_/LA_above_ is the proportion between the leaf area below the sensor in relation to the leaf area above the sensor (in m^−2^ tree^−1^).

### Tree Biomass Compartment Measurement

Loss in leaf biomass was assessed once a week as follows. We collected and carefully removed already brown needles from the branches of each individual tree. Leaves were oven-dried at 60°C for 48 h, and the dry weight was measured. Total tree biomass was determined at the end of the experiment and separated into needles, wood, and roots (Supplementary Table 1). We separately assessed leaf biomass above and below the sap flow sensor in order to derive total tree transpiration (see section Soil Water Content and Transpiration). As needle elongation was finished before the experiment started, initial leaf biomass per tree was considered as the sum of the remaining needles at the end of the experiment plus leaf biomass shed during the experiment, assuming a constant above-sensor leaf biomass/below-sensor biomass ratio. In order to obtain the actual daily leaf biomass time series, we linearly interpolated cumulative leaf shedding between measurement campaigns and subtracted it from the initial leaf biomass. For each tree, conversion from leaf biomass to leaf area was done based on specific leaf area (SLA, cm^2^ g^−1^), obtained by measuring the width and the length of a representative sub-sample per tree (in total *n* = 18). No significant differences were detected between monitored and not-monitored individuals regarding SLA and tree biomass in different compartments (see Supplementary Table 1).

### Xylem Loss in Hydraulic Conductance

We measured xylem vulnerability of branches using the Cavitron technique (Cochard, 2002). Briefly, the centrifugal force of the Cavitron increases the negative pressure in the stem while the hydraulic conductivity and the loss thereof is measured concurrently. At the beginning of the experiment, we sampled the terminal part of the lowest branch (~33 cm long) in five randomly selected non-monitored trees. Branches were tightly wrapped in cling film and additionally sealed in plastic bags before being transported to Innsbruck where they were kept at 4°C for 2 days. Samples were prepared for the Cavitron as follows: first, side twigs and needles were removed, and branches were re-cut under water several times to relax xylem tension, until a sample length of ~28 cm was reached and debarked at both ends (~5 cm) to avoid clogging of tracheids by resin. The cut and debarked ends were cleaned with a sharp razor blade. To remove native embolisms *via* vacuum infiltration, submerged samples were subjected to a low-pressure water flow (0.08 MPa) for 30 min with distilled, filtered (0.22 μm), and degassed water containing 0.005% (v/v) Micropur. Then, branch segments were fixed into a custom-made, honeycomb 28-cm rotor and positioned in a Sorvall RC-5 centrifuge (Thermo Fisher Scientific, Waltham, MA, United States). Distilled, filtered (0.22-μm pore size), and degassed water with 0.005% (v/v) Micropur water purifier (Katadyn Products, Wallisellen, Switzerland) preventing microbial growth, was used for the measurements. Percent loss of conductivity (PLC, in %) measurements started at a force of about −0.5 MPa, which was gradually increased until minimum xylem hydraulic conductivity (*K*_*xylem*_, mol m^−1^ s^−1^ MPa^−1^) was reached. PLC was recorded at about −0.5 MPa pressure increase steps. We assumed that losses in conductivity reflect losses in xylem conductance (*k*_*xylem*_, in mol m^−2^ s^−1^ MPa^−1^). In order to model normalized *k*_*xylem*_ (*k*_*norm*_, 0–1) responses to xylem water potential increase (Ψ_*xylem*_), we evaluated three different response functions: a Weibull function (WB), a sigmoid exponential (SE) function, and the original SOX model function (SOXf)

(2) $$\begin{array}{l} k_{norm,WB}=1-\left(1-e^{-{\left(\frac{{\text{Ψ}}_{xylem}}{{\text{Ψ}}_{ref}}\right)}^{A_{coef}}}\right) \end{array}$$

(3) $$\begin{array}{l} k_{norm,SE}=1-\frac{1}{\left(1+(\exp^{A_{coef}\;(\Psi_{xylem}-\Psi_{50})}\right)} \end{array}$$

(4) $$\begin{array}{l} k_{norm,SOXf}=\left(\frac{1}{1+{{\left(\frac{{\text{Ψ}}_{xylem}}{{\text{Ψ}}_{50}}\right)}^{A_{coef}^{\;}}}^{\;}}\right) \end{array}$$

where *k*_*norm, WB*_, *k*_*norm, SE*_, and *k*_*norm, SOXf*_ are the predicted normalized *k*_*xylem*_ of theWB, SE, and SOXf formulations, respectively. Ψ_xylem_ (MPa) is the xylem water potential. In WB, Ψ_ref_ is the reference xylem water potential (MPa) when *k*_*norm, WB*_ is equal to exp^(−1)^ ~0.37. In SE and SOXf, Ψ_50_ is the xylem water potential at which *k*_*norm*_ = 0.5. In all the three formulations, A_coef_ is a unit-less shaping parameter. See section Xylem Vulnerability for details about the fitting procedure.

### Photosynthesis *A_*n*_*/*C_*i*_* Curves

In order to provide estimates for photosynthetic parameters in the FvB model, *A*_*n*_/*C*_*i*_ curves were measured on trees grown in the greenhouse in June, 2019. Measurements were made on sun-exposed needles, using a portable infrared gas analyzer system (LI-6800, Li-Cor, Inc., Lincoln, NE, United States) with a 6-cm^2^ leaf chamber. The measurements were taken at saturating PAR (PAR ≥ 1,200 μmol m^−2^s^−1^). Assimilation in relation to [CO_2_] in the intercellular space (*A*_*n*_/*C*_*i*_) curves was generated by increasing atmospheric CO_2_ concentration (C_a_) inside the chamber in five steps, starting at ~400 μmol mol^−1^ and then progressively increasing C_a_ by ~200 μmol mol^−1^ each step, up to a maximum of ~1,200 μmol mol^−1^. Leaf temperature (Tleaf, in °C) and other standard variables were also measured. These measurements were taken under prevailing leaf temperature (~25°C) and humidity conditions within the greenhouse. A total of 19 *A*_*n*_/*C*_*i*_ curves were measured this way. We used these data to determine carboxylation velocity limited by rubisco activity at 25°C (*V*_*cmax*, 25_ in μmol m^−2^ s^−1^) and the carboxylation velocity limited by RuBP regeneration rate (*J*_*max*,25_ in μmol m^−2^ s^−1^).

### Modeling Approach

**Figure 2:**
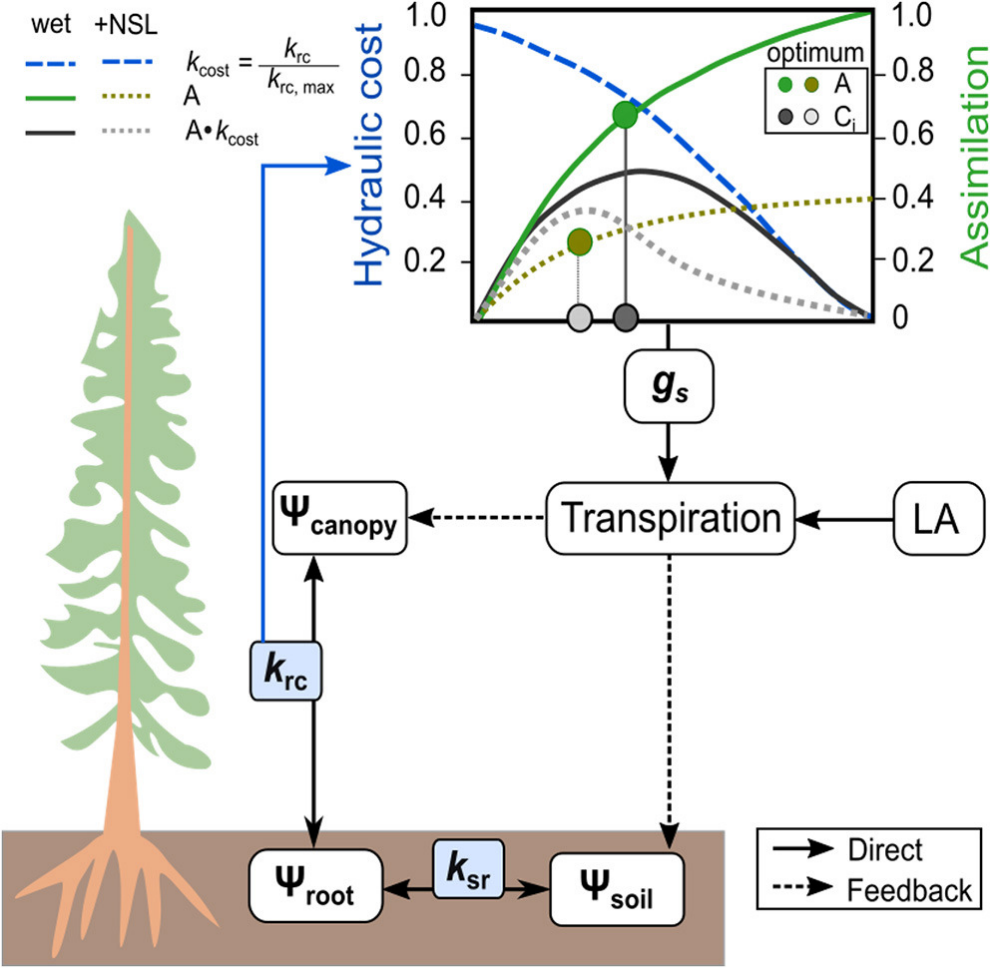
Scheme of the SOX+ model. Soil-to-atmosphere is described as a hydraulic pathway considering three nodes: hydraulic flow from soil to the roots, mediated by soil-to-root conductance (k_sr_), hydraulic flow from roots to the canopy, mediated by root-to-canopy conductance (k_rc_), and hydraulic flow from the canopy to the atmosphere, mediated by stomatal conductance (gs). The optimum gs is determined by considering the photosynthetic gain (A) multiplied by the hydraulic cost function (k_cost_), which describes the decreases in k_rc_ as Ψ_*canopy*_ becomes more negative. Transpiration is calculated at the tree level by multiplying gs and leaf area, and it has a direct effect on canopy water potential, an indirect effect on soil water content, and therefore on Ψ_*soil*_. Non-stomatal limitations of photosynthesis (NSL) decrease optimum stomatal conductance because of limiting A when Ψ_*canopy*_ declines below a predefined threshold.

The tree hydraulic modeling approach is based on the process-based model SOX (Eller et al., 2018, 2020). We have modified the photosynthetic module, added the possibility to address non-stomatal-limitations of photosynthesis (see below), and included a third conductance node (i.e., the soil-to-root hydraulic conductance, Figure 2). In the following, we refer to the modified model version as the SOX+ model. Briefly, the model operates on the assumption that plants regulate stomatal conductance to maximize photosynthesis, while minimizing the loss in root-to-canopy water conductance. The model is relatively simple in terms of parameterization and computational power required and, hence, applicable for a wide range of ecosystem models. The environmental drivers required as an input to run the model are air temperature, PAR, soil water content, and air relative humidity. Here, we describe the main processes involved such as the modification for SOX+, while a more in-depth description of the original SOX model and the analytical solutions for its equations can be found in Eller et al. (2020).

Water flow within the tree is described as a three-step pathway with three different hydraulic conductance nodes: the soil-to-root hydraulic conductance (*k*_*sr*_,mol m^−2^ s^−1^MPa^−1^), the root-to-canopy hydraulic conductance (*k*_*rc*_, mol m^−2^ s^−1^MPa^−1^), and the leaf-atmosphere hydraulic conductance (*g*_*s*_, mol m^−2^ s^−1^). The model assumes a steady state of tree water status, i.e., it does not account for tree capacitance. The soil-to-root conductance *k*_*sr*_ is calculated following Campbell (1985):

(5) $$\begin{array}{l} k_{sr}=k_{sr,\max}*{\left(\frac{SWC}{SWC_{FC}}\right)}^{\left(2+\frac{3}{b}\right)} \end{array}$$

where *k*_*sr,max*_ is the *k*_*sr*_when SWC is at field capacity, SWC is the volumetric soil water content (m^3^ m^−3^), SWC_FC_ is the volumetric SWC at field capacity (m^3^ m^−3^), and *b* is an empirical coefficient depending on average soil particle size characteristic, calculated as described in Campbell (1985). Using k_sr_ on the one hand, and transpiration per unit of leaf area (E, mol m^−2^ s^−1^) on the other, the model calculates the water potential within the roots (Ψ_*root, t*_) based on Darcy's law at hourly time-steps as

(6) $$\begin{array}{l} {\text{Ψ}}_{root,t}={\text{Ψ}}_{soil,t}-\frac{E_{\left(t-1\right)}}{k_{sr\left(t-1\right)}} \end{array}$$

where Ψ_*soil,t*_ is the soil water potential at time step *t* (in MPa), calculated according to Campbell (1985) from soil physical properties. Canopy water potential (Ψ_*canopy,t*_) is closely linked to root water potential, which in turn strongly depends on soil water availability. We assume a simple gravimetrical decline with height, neglecting a potential influence of stored plant water:

(7) $$\begin{array}{l} {\text{Ψ}}_{canopy,t}={\text{Ψ}}_{root,t}-\frac{E_{\left(t-1\right)}}{k_{rc\left(t-1\right)}}-h\rho g*10^{-6} \end{array}$$

where *k*_*rc*(*t*−1)_ is the hydraulic conductance from the roots to the canopy at t – 1 (mol m^−2^ s^−1^MPa^−1^) based on the canopy water potential at that time. Therefore, we assume that Ψ_*canopy, m*_ equals Ψ_*xylem*_ and that *k*_*rc*_(Ψ_*canopy,m*_) follows the shape of *k*_*norm*_(Ψ_*xylem*_), multiplied by the maximum root-to-canopy hydraulic conductance (*k*_*rc, max*_, in mol m^−2^s^−1^ Mpa^−1^). For representing *k*_*norm*_(Ψ_*xylem*_), we selected Equation 2, as we found it represented measured data best (see section Leaf Shedding Speed Increased Concurrently With Transpiration Stop). Further, *h* is the plant height (in m), ρ is the density of water (997 kg m^−3^), and *g* is gravity (9.8 m s^−2^). The 10^−6^ multiplier transforms Pa to MPa. For each day, we calculated pre-dawn canopy water potential (Ψ_*canopy, PD*_, MPa) using equation 7 but assuming E_(t−1)_ ≈ 0, which results in Ψ_*canopy, PD*_ being dependent on Ψ_*soil*_ minus the gravimetric component. As in the previous SOX model iterations (Eller et al., 2018, 2020), SOX+ represents the water potential in the xylem with a dampened canopy water potential (Ψ_*canopy,m*_, MPa), calculated as $\frac{{\text{Ψ}}_{canopy,t}+{\text{Ψ}}_{canopy,PD}}{2}$ for all the hourly calculations to account for the gradual decline in water potential along the plant hydraulic pathway. Such assumption greatly simplifies the calculation of water potential drop within the tree (e.g., Sperry and Love, 2015; Sperry et al., 2017).

The core assumption in SOX+ is that stomatal conductance (gs, mol m^−2^ s^−1^) is regulated to maximize photosynthetic net assimilation (*A*_*n*_, μmol m^−2^ s^−1^) while minimizing the decrease in xylem hydraulic conductance, represented by root-to-canopy conductance (*k*_*rc*_) here. Eller et al. (2020) solved for *gs* using an analytical approximation based on the partial derivatives of *A*_*n*_ with respect to CO_2_ concentration in the chloroplast (C_i_, μmol mol^−1^) and *k*_*rc*_ with respect to Ψ_*canopy,m*_.

(8) $$\begin{array}{l} gs_{SOX}=\;0.5\frac{\partial A_{n}}{\partial C_{i}}\left(\sqrt{\left(\frac{4\xi }{\partial A_{n}/\partial C_{i}}+1\right)}-1\right) \end{array}$$

where δ*A*_*n*_*/*δ*C*_*i*_ represents the gain in net photosynthesis per unit of *C*_*i*_ increase, i.e., the positive effect of opening the stomata on *A*_*n*_, solved numerically as in Eller et al. (2020), while ξ represents the cost in terms of loss in hydraulic conductance of opening the stomata as canopy water potential declines and/or vapor pressure deficit increases (Equation 9). Specifically, the lower the ξ, the lower the stomatal conductance projected by SOX+. If estimates of gs_SOX_ are lower than a predefined minimum leaf conductance, representing leaf leakiness once stomata are fully closed (g_min_, in mmol m^−2^ s^−1^; here 2 mmol m^−2^ s^−1^), we considered gs equal to g_min_, otherwise gs = gs_SOX_, following Duursma et al. (2019). Note that in the approach, g_min_ integrates leaf water losses both because of imperfect stomatal closure and leaf cuticular conductance, considering a well-coupled canopy and low wind speed conditions (e.g., Cochard et al., 2021).

(9) $$\begin{array}{l} \xi \;=\;\frac{2}{\frac{1}{k_{norm,rc}}*\frac{\delta k_{norm,rc}}{\delta {\text{Ψ}}_{canopy,m}}*rplant_{\min,\text{ Ψ}}*1.6*VPD} \end{array}$$

(10) $$\begin{array}{l} rplant_{\min,\text{Ψ}}=\frac{rplant_{\min}}{k_{norm,r,c}\left({\text{Ψ}}_{canopy,m}\right)} \end{array}$$

where VPD is the vapor pressure deficit at leaf level (kPa) and $\frac{\delta k_{norm,rc}}{\delta {\text{Ψ}}_{canopy,m}}$ represents the decrease in conductance as mean canopy water potential increases, solved numerically as described in Eller et al. (2020). *rplant*_, *min*_ is the minimum plant resistance (in MPa m^2^ s mol^−2^), a parameter used to describe the increase in whole tree resistance with decreasing Ψ_*canopy,m*_.

Net assimilation (*A*_*n*_) is calculated according to the Farquhar, von Caemmerer, and Bell photosynthesis model (FvCB, Farquhar et al., 1980; De Pury and Farquhar, 1997). Briefly, the FvCB assumes that *A*_*n*_ is limited by either rubisco carboxylation velocity (_Avc_) or RuBP regeneration (*A*_*j*_), and is calculated considering dark respiration (R_d_). All of these processes depend on leaf temperature (Farquhar et al., 1980; Harley et al., 1992; Bernacchi et al., 2001), which we assume to equal air temperature.

(11) $$\begin{array}{l} A_{n}=argmin\left(A_{vc},A_{j}\right)-R_{d} \end{array}$$

As novelty compared with the original SOX model, SOX+ accounts for non-stomatal limitations (NSL) in photosynthesis, as have been repeatedly found important to properly represent reductions of transpiration and productivity under drought stress (e.g., Keenan et al., 2010; Duursma and Medlyn, 2012; Drake et al., 2017; Yang et al., 2019, Gourlez de la Motte et al., 2020). NSL assume that additional constrains on *A*_*n*_ arise with decreasing Ψ_*canopy*_ from mesophyll conductance reductions, biochemical limitations, or other indirect effects (e.g., Fatichi et al., 2014). Here, note that in SOX+ and according to Equation 8, NSL will result in a reduction in δ*An/*δ*C*_*i*_, thus also indirectly leading to reduced gs_SOX_. In SOX+, declining Ψ_*canopy,m*_[*f* (Ψ_*canopy,m*_), unit-less, its value ranging between 1 (no limitation) and 0 (complete limitation)] is calculated based on Tuzet et al. (2003) as follows:

(12) $$\begin{array}{l} f\left({\text{Ψ}}_{canopy,m}\right)=\left(\frac{1+\;\exp^{\left(a_{Tuz}^{*}{\text{Ψ}}_{ref,Tuz}\right)}}{1+\;\exp^{\left[a_{Tuz}^{*}\left({\text{Ψ}}_{ref,Tuz}-{\text{Ψ}}_{canopy,m}\right)\right]}}\right) \end{array}$$

where Ψ_*ref,Tuz*_ and *a*_*Tuz*_ are two empirically determined parameters, Ψ_*ref,Tuz*_ being a reference canopy water potential (in MPa), as defined in Tuzet et al. (2003), and *a*_*Tuz*_ being a unit-less coefficient that determines the sensitivity of the sigmoid curve to Ψ_*canopy,m*_reductions. After simulating *f* (Ψ_*canopy,m*_), apparent V_cmax_ and apparent J_max_ are computed as

(13) $$\begin{array}{l} X_{apparent}=X_{\max,25}^{*}f\left({\text{Ψ}}_{canopy,m}\right) \end{array}$$

where the X_apparent_ (μmol m^−2^ s^−1^) is the apparent kinetic parameter value for either V_cmax_ or J_max_ for a X_max,25_ (μmol m^−2^ s^−1^) reference value (see **Table 2**) multiplied by the NSL (Equation 12), represented by the unit-less stress term *f* (Ψ_*canopy,m*_). These new kinetic parameters accounting for direct impacts of Ψ_canopy_ decline on photosynthesis are then used to calculate *A*_*vc*_ and *A*_*j*_ in Equation 11.

Because of the feedback mechanism between *A*_*n*_ and gs, SOX+ solves both processes iteratively for the equilibrium C_i_.

### Model Parameterization and Calibration

#### Photosynthesis in the FvCB Model

**Figure 3:**
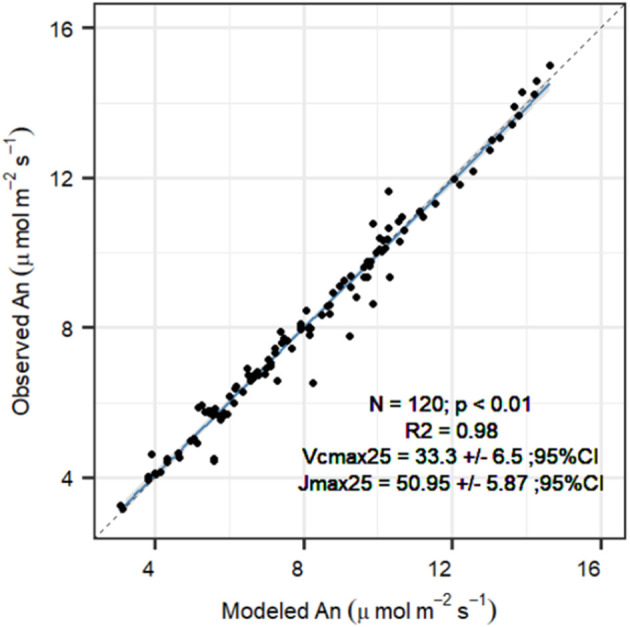
Comparison between FvCB model net assimilation (An, μmol m^−2^ s^−1^) projections with observations after parameter optimization for each *A*_*n*_/*C*_*i*_ curve (*n* = 19), for the *P. sylvestris* saplings grown in the greenhouse in year 2019. Reported are the fit (*R*^2^), the median optimum *V*_*cmax*,25_ ± 95% confidence interval, and the median optimum *J*_*max*,25_ ± 95% confidence interval. The solid line indicates the trend between observed and modeled An (*p* < 0.01), with the gray area representing the 95% CI. The slope was not significantly different than 1 (*p* > 0.1), and intercept was not significantly different than 0 (*p* > 0.1) based on a *t*-test. Dashed line represents the 1:1 fit.

Temperature dependencies of rubisco kinetics were obtained from Bernacchi et al. (2001). Temperature dependencies for the FvCB model were obtained for *P. sylvestris* from Wang et al. (1996), see Table 1 for the parameter values. To obtain the carboxylation velocity limited by rubisco activity at 25°C (*V*_*cmax*, 25_ in μmol m^−2^ s^−1^) and the carboxylation velocity limited by RuBP regeneration rate (*J*_*max*,25_ in μmol m^−2^ s^−1^), we used measured *A*_*n*_*/C*_*i*_ curves under no drought stress to calibrate the FvCB model for each curve individually, using the package “DEOptim” (Mullen et al., 2011). The algorithm obtains the most likely parameters providing the observations, a parameter distribution and a likelihood function, by performing differential evolution optimization (see below for more details). We used the default three-chain settings to establish non-informative priors within the biological meaningful range (Table 2). The objective function for optimization was a Gaussian log-likelihood distribution. This provided a *V*_*cmax*, 25_ and a *J*_*max, k*25_ value for each curve. To summarize the average photosynthesis kinetics of the *P. sylvestris* population, we used the median calibrated values of *V*_*cmax*, 25_ and *J*_*max*,25_ to run the SOX+ model (Table 2). We calculated the dark respiration rate at 25°C (*R*_*d*, 25_ in μmol m^−2^ s^−1^) as *R*_*d*, 25_ = 0.015^*^V_*cmax*, 25_, according to Collatz et al. (1991). After FvCB model optimization, *A*_*n*_estimates were very close to observations [Figure 3, slope not significantly different than 1(*p* > 0.1) and intercept not significantly different than zero (*p* > 0.1)]. The median *J*_*max*,25_ to *V*_*cmax*, 25_ ratio was ~1.7, with *V*_*cmax*, 25_ values of 33.3[27.7–38.9] μmol m^−2^ s^−1^ (median [95% CI]), and *J*_*max*,25_ values of 51[45.1–56.8] μmol m^−2^ s^−1^ (Table 2). To run the SOX+ model, we used the median values of *V*_*cmax*, 25_ and *J*_*max*,25_.

**Table 1:** Fixed, not previously calibrated parameters used in the SOX+ model.

**Parameter**	**Units**	**Value**	**Source**
EaV_cmax_	J mol^−1^	52,750	Wang et al. (1996)
EdV_cmax_	J mol^−1^	202,600	Wang et al. (1996)
SV_cmax_	J mol^−1^ K^−1^	669	Wang et al. (1996)
EaJ_max_	J mol^−1^	61,750	Wang et al. (1996)
EdJ_max_	J mol^−1^	185,600	Wang et al. (1996)
SJ_max_	J mol^−1^K^−1^	621	Wang et al. (1996)
Rd_25_	μmol m^−2^ s^−1^	0.5	Calculated as V_cmax, 25_ * 0.015
Q_10_	unitless	2	This study
$\Gamma_{25}^{*}$	μmol mol^−1^	42.2	Bernacchi et al. (2001)
EaΓ^*^	J mol^−1^	37,830	Bernacchi et al. (2001)
K_c, 25_	μmol mol^−1^	404	Bernacchi et al. (2001)
EaK_c_	J mol^−1^	84,200	Bernacchi et al. (2001)
K_O, 25_	μmol mol^−1^	278,000	Bernacchi et al. (2001)
EaK_O_	J mol^−1^	15,200	Bernacchi et al. (2001)
g_min_	mmol m^−2^ s^−1^	2	This study
LA	m^−2^	1.35	This study
Height	M	2	This study
SoilDensity	g cm^−3^	1.3	This study
FieldCapacity	cm^−3^cm^−3^	0.35	This study

EaV_cmax_, EdV_cmax_, and SV_cmax_ are the activation energy, deactivation energy, and entropy for the V_cmax_ temperature response, respectively. EaJ_max_, EdJ_max_, and SJ_max_ are the activation energy, deactivation energy, and entropy for the J_max_ temperature response. Rd_25_ is the dark respiration at 25°C, calculated from V_cmax, 25_ according to Collatz et al. (1991), and Q_10_ is the increase rate of R_d_ as temperature increases. $\Gamma_{25}^{*}$ and EaΓ^*^ are the CO_2_ compensation point at 25°C and the activation energy of the compensation point. K_O, 25_ and K_c, 25_ are the kinetic constants of oxidase and carboxylase activity for rubisco at 25°C, respectively. EaK_O_ and EaK_c_ are the energy activation for the oxidase and carboxylase activity of rubisco, respectively. g_min_ is the minimum leaf conductance, which integrates both residual stomatal conductance because of imperfect stomatal closure and leaf cuticular conductance. LA is the average tree leaf area on day 1 of the experiment. Height is the average tree height. Soil density and field capacity are the dry soil weight per unit of soil volume and the volumetric soil water content at saturation, respectively. Given are parameter names, units, values, and reference.

**Table 2:** Results of the three-step SOX+ model parameterization.

				**Prior**		**Parameters (** ***n*** **=** **19)**					
**Model**	**Parameter**	**Units**	**Distribution**	**Min**	**Max**	**Median**	**95%CI**				
**FvCB (Optimization)**	*V_*cmax*, 25_*	μmol m^−2^ s^−1^	Uniform	10	80	33.3	[26.8–38.8]				
	*J_*max*,25_*	μmol m^−2^ s^−1^	Uniform	30	140	51	[45.1–56.8]				
*k_*xylem*_ (Ψ_*xylem*_),_*WB*_*				**Prior**	**Combined posterior (** ***n*** **=** **5)**						
**(Calibration)**	**Parameter**	**Units**	**Distribution**	**Min**	**Max**	**Q2.5%**	**Median**	**Q97.5%**			
	*Ψ_*ref*_*	MPa	Uniform	−1.5	−3.5	−3.21	−3.01	−2.12			
	*A_*coef*_*	unitless	Uniform	1	8	2.03	2.85	3.16			
				**Prior**		**Posterior (Hydraulic** **+** **NSL)**			**Posterior (Hydraulic)**		
SOX+ (Calibration)	**Parameter**	**Units**	**Distribution**	**Min**	**Max**	**Q2.5%**	**Median**	**Q97.5%**	**Q2.5%**	**Median**	**Q97.5%**
	*k_*sr,max*_*	mol m^−2^ s^−1^ MPa^−1^	Uniform	0.01	1	0.36	0.77	0.99	0.01	0.01	0.01
				Mean	SD						
	*k_*rc, max*_*	mol m^−2^ s^−1^ MPa^−1^	Gaussian	0.025	0.01	0.024	0.035	0.043	0.033	0.042	0.045
	*Rplant_*min*_*	m^2^ s MPa mol^−1^	Gaussian	10	3	4.75	4.87	4.92	4.75	4.77	4.8
	*Ref_*Tuz*_*	MPa	Gaussian	−1.5	0.5	−1.75	−1.72	−1.71	NA	NA	NA
	*A_*Tuz*_*	unitless	Gaussian	3	0.5	2.75	2.92	2.97	NA	NA	NA

*For the FvCB photosynthesis model, the prior assumed for each individual A_n_/C_i_ curve (n = 19) for V_cmax, 25_ and J_max, 25_ is provided, together with the median and 95%CI parameter value calculated from the 19 individual optimizations. For the Weibull (WB) xylem vulnerability model kyxlem(Ψ_xylem_),_WB_ calibration, the prior parameter distribution assumed for each individual curve (n = 5) is provided for Ψ_ref_ and A_coef_ parameters. The median and the 95% credible interval are reported from the combined posterior distribution for the five curves. Two different SOX+ model calibrations were performed, one accounting for non-stomatal limitations (NSL, Hydraulic + NSL) and one not accounting for NSL (Hydraulic). SOX+ model calibrations are reported as median posterior parameter estimates ± 95% credible interval for the five (three) parameters included in the calibration for the Hydraulic + NSL (Hydraulic). For each model and parameter, assumed prior parameter distributions are also reported*.

#### Xylem Vulnerability

In order to decide for an appropriate response function and define the respective parameters describing *k*_*xylem*_(Ψ_*xylem*_), we first inversely calibrated each of the proposed equations (Equations 2–4) with the *k*_*xylem*_(Ψ_*xylem*_) observations. Independent of the equation we proceeded as follows: we calibrated one vulnerability curve per sample using each of the three equations, using broadly set but biologically meaningful priors (Table 2) and a Gaussian likelihood function. The sampler used was a differential evolution Markov Monte-Carlo Chain (MCMC) with memory and a snooker update (DEzs, terBraak and Vrugt, 2008), implemented in the “BayesianTools” R package (Hartig et al., 2017). We ran each calibration for 50 k iterations, and then the first 30 k where discarded as a burn-in. For initial trials, we ran tree calibrations for each vulnerability curve and addressed between-chain convergence *via* Gelman-Rubin convergence diagnosis (Gelman and Rubin, 1992). As running the model this way is time-consuming and the initial trials provided very low Gelman–Rubin scores for all the three equations (G-R < 1.02, *n* = 2 for each model), which indicates fast convergence, we visually inspected the MCMC to confirm convergence afterward. Once all individual curves were calibrated, we merged the five posteriors to obtain the average response by sampling with replacement 1 k times the posterior distribution for each curve. We then merged the samples together into a new combined posterior distribution. From this combined posterior distribution, we obtained the median parameter value ±95% confidence intervals. Model predictions were performed by sampling 1 k samples from the combined posterior. Goodness of fit was assessed for each run through the root mean square error (RMSE) and a pseudo-*R*^2^ calculated from Spearman's correlation coefficient as pseudo-*R*^2^ = [cor (Observed, Modeled)]^2^. Finally, SOX+ was run with the median parameters of the equation that presented the best fit (i.e., the Weibull equation, Equation 2).

#### SOX+ Model

We calibrated the SOX+ model based on average daily transpiration rates of the tree population (n = 6). To do so, we performed Bayesian inverse model calibration (e.g., Ellison, 2004; Hartig et al., 2012) selecting the parameters *k*_*sr,max*_, *k*_*rc,max*_, *rplant*_*min*_, Ψ_*ref,Tuz*_, and *a*_*Tuz*_. Since we were primarily interested in the initial transpiration response to decreasing soil water content to see if SOX+ was able to capture the dry-down phase, we only used the initial 19 days (DOY 212–230) for calibration, further excluding days 216 and 217 because of low VPD and PAR conditions in the greenhouse (Figure 1). As including or not a given process changes the whole structure of the model, and because we wanted to run SOX+ with and without accounting for NSL, we performed two different calibrations (hereafter referred as “Hydraulic,” i.e., SOX + model without including NSL, and “Hydraulic+NSL,” i.e., SOX + model including NSL). The procedure was the same in both cases, though the “Hydraulic” calibration did not include the Ψ_*ref,Tuz*_and *a*_*Tuz*_ parameters. Again, we used broadly set, biologically meaningful priors (Table 2), a Gaussian likelihood function, and the differential evolution MCMC with memory and a snooker update (DEzs, terBraak and Vrugt, 2008) as implemented in the “BayesianTools” R package (Hartig et al., 2017). We ran each calibration three times, 30 k iterations each, and then we discarded the first 20 k iterations as a burn-in. Between-MCMC convergence was addressed by the Gelman–Rubin convergence diagnosis (G-R <1.1 for both calibrations, G-R <1.1 considered being a conservative threshold; Brooks and Gelman, 1998). To assess the calibrated SOX+ performance, we ran for 500 times both the “Hydraulic” and “Hydraulic+NSL” model formulations for the entire time series (DOY 212–265), by sampling resulting posterior parameter distribution. For each run, we compared projected daily cumulated transpiration with the observations using a linear model accounting for temporal autocorrelation with an auto-regressive [corAR()] correlation structure centered in DOY. This was done with the package “nlme” (Pinheiro et al., 2021). Goodness of fit was assessed through the RMSE and a pseudo-*R*^2^ [${\text{R}}_{\text{cs}}^{2}$; Cox and Snell, 1989), based on the deviance from the regressive model with respect to the null model and reported as the median RMSE and ${\text{R}}_{\text{cs}}^{2}$ for the 500 runs.

### Simulation Scenarios

#### NSL and Leaf Shedding Importance in Preventing Loss in Hydraulic Conductance

To quantify the importance of non-stomatal limitations (NSL) of photosynthesis and leaf shedding in preventing *k*_*rc*_ decline, we ran the calibrated SOX+model for 54 days (DOYs 212–265) under the following scenarios: (1) hydraulic limitations with observed leaf shedding dynamics, i.e., accounting for the daily leaf area reduction (Hydraulic scenario, ran with the “Hydraulic” calibration); (2) Hydraulic limitations and NSL, but considering leaf area constant at the initial value during the entire simulation (Hydraulic + NSL scenario, ran with the “Hydraulic+NSL” calibration); and (3) Hydraulic limitations and NSL plus observed leaf shedding dynamics (Hydraulic + NSL + leaf shedding scenario, ran with the “Hydraulic+NSL” calibration). This setup enabled to assess the importance of NSL and leaf shedding on *k*_*rc*_loss separately. To obtain model projections for each scenario, we sampled 500 times the posterior parameter distribution. We report the daily evolution of percent loss in *k*_*rc*_(PLk_rc_, in %), calculated as 100^*^k_*rc*_(Ψ_*canopy, PD*_)/*k*_*rc, max*_, as the median ± 95% CI of the 500 runs.

#### Importance of Leaf Shedding at Different Degrees of Leaf Leakiness

We tested the sensitivity of the model to variations in minimum leaf conductance (g_min_). This highly uncertain parameter is assumed to vary strongly between plants, while being extremely important for water loss and leaf shedding under extreme drought stress (e.g., Vilagrosa et al., 2003; Hochberg et al., 2017; Duursma et al., 2019; Li et al., 2020). It is assumed that plants with higher g_min_ will likely benefit more from leaf shedding, as the reduction in water loss per unit of leaf shed will be higher. The model formulation “Hydraulic+NSL” was run with two different g_min_ values (g_min_ = 2 mmol m^−2^ s^−1^ and g_min_ = 3 mmol m^−2^ s^−1^, a 50% increase), considering either constant leaf area, i.e., no leaf shedding, or observed leaf dynamics, i.e., observed leaf shedding. Again, posterior parameter sampling with replacement was carried out for 500 model runs for each of the 2 × 2 scenarios (leaf dynamics × g_min_ assumption). Then, for each g_min_ scenario, we calculated the daily average cumulative benefits of leaf shedding as

(14) $$\begin{array}{l} Benefit_{LS,i}=\;\frac{\overset{i}{\underset{i=1}{\sum }}PLk_{rc,NoShed,i}-PLk_{rc,Shed,i}}{i} \end{array}$$

where *Benefit*_*LS,i*_ is the daily average cumulative reduction in percent loss of *k*_*rc*_ if trees dynamically shed their leaves relative to the same trees in the absence of leaf shedding, “i” is the day since the beginning of the experiment, and *PLk*_*rc,NoShed,i*_ and *PLk*_*rc,Shed,i*_ are the percent loss in *k*_*rc*_ on day “i” in the absence or presence of leaf shedding, respectively. For this and all the other statistical analyses described before, as well as to develop and implement the SOX+ model, we used the R statistical program (R Core Team, 2021, Version 3.6.1).

## Results

### Calibration of Xylem Vulnerability Models

**Figure 4:**
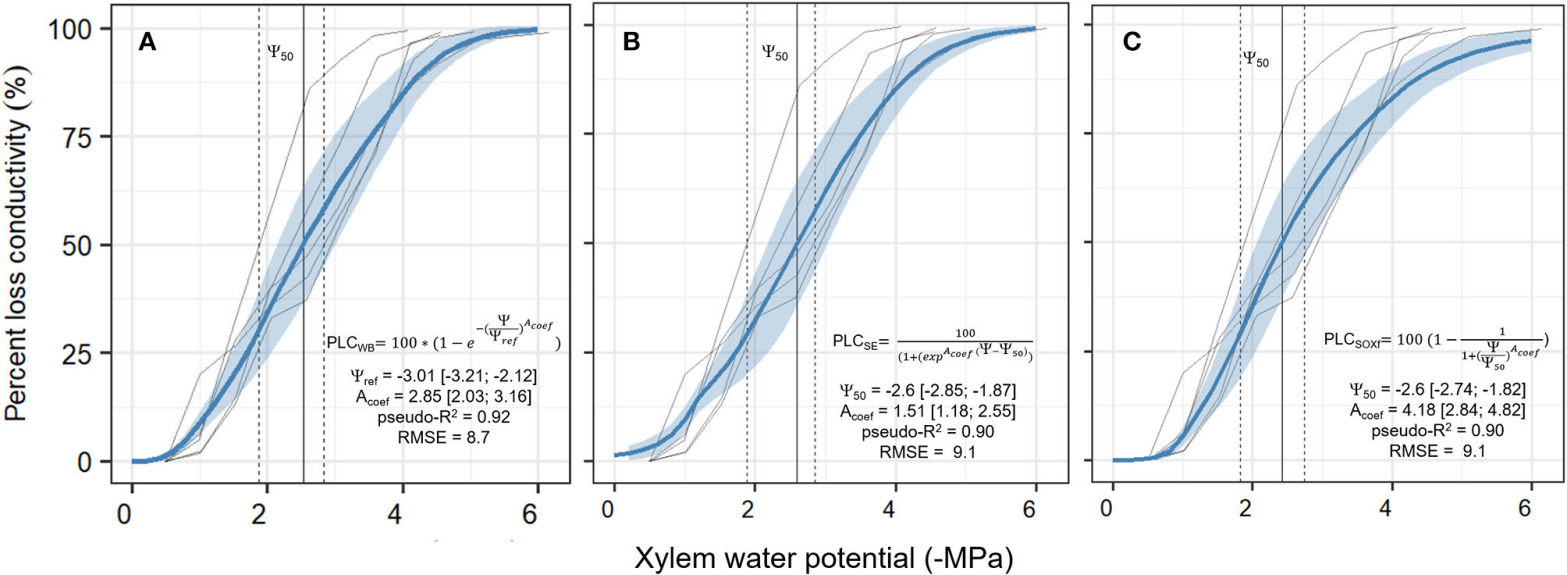
Calibration of the three different xylem vulnerability models [Weibull model (WB, **A**), the Sigmoid Exponential (SE, **B**), and the original SOX formulation (SOXf, **C**)] for the *P. sylvestris* saplings. Solid transparent lines are the five measured curves of the observed percent loss in xylem conductivity (PLC, in %) in relation to xylem water potential decrease (Ψ_*xylem*_, in -MPa). Solid blue line ± shadowed blue area is the median ± 95 CI obtained from 1 k model runs sampling the combined posterior parameter estimate, resulting from curve-individual calibrations for each model, as described in section Xylem Vulnerability. The vertical solid line shows the Ψ_50_ value; this is when xylem has lost 50% of its conductivity (±95% CI, vertical dashed lines). For each model, formulation and median ± 95% CI values for each parameter are provided. Note that in the WB model Ψ_ref_ parameter meaning is not equivalent to the Ψ_50_ parameter from the other two models. Goodness of fit is given as the median pseudo-*R*^2^ and the median root mean square error (RMSE) of all model runs.

All the three equations to describe hydraulic vulnerability [Weibull (WB); sigmoid exponential (SE), and original SOX+ formulation (SOXf)] provided similar, overlapping results. The median xylem water potentials at which 50% of loss in hydraulic conductivity occurred were: WB −2.53 MPa, SE −2.6 MPa, and SOXf−2.6 MPa. The WB function described the observed trends slightly better than the other functions while also maintaining the assumption in the original SOX model that loss of hydraulic conductivity does not occur at Ψ_*xylem*_ values ≈ 0 (Figure 4). Therefore, WB was selected to run SOX+ for *P. sylvestris* in this experiment. Parameter values describing prior and the posterior parameters are listed in Table 2.

### Leaf Shedding Speed Increased Concurrently With Transpiration Stop

**Figure 5:**
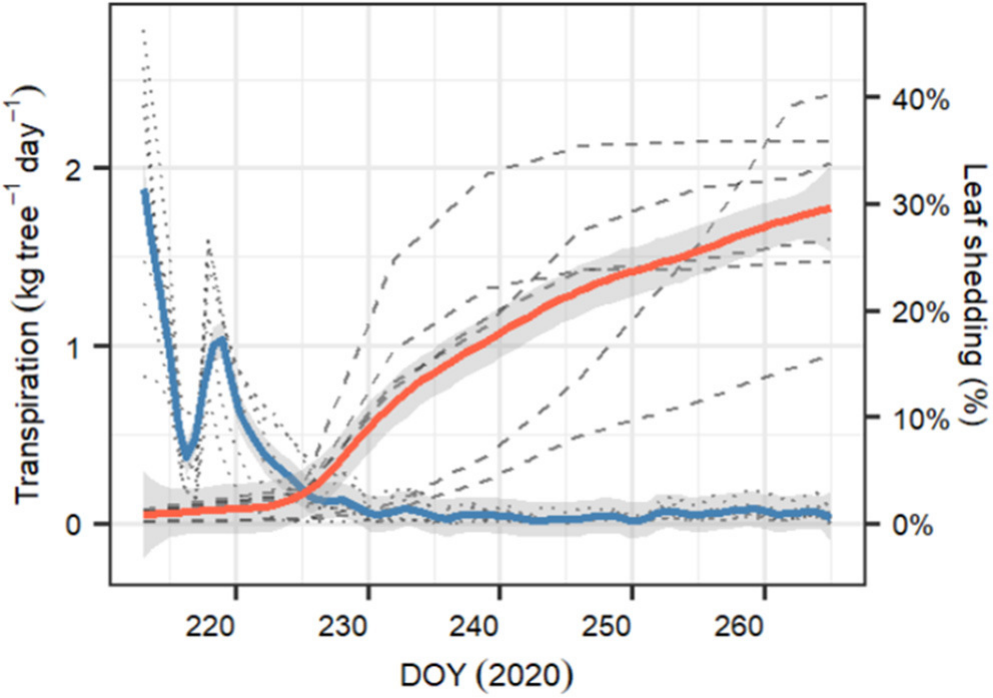
Temporal evolution of whole-tree transpiration (in kg tree^−1^ day^−1^) and percent of leaf shed for the six monitored *P. sylvestris* saplings growing in the greenhouse during the whole length of the experiment (DOY 212–265). Blue line represents the average daily transpiration ±1 SD, whereas red line represents the daily cumulated percent of leaf shed since the beginning of the experiment ±1 SD. Transparent dotted lines are the observed daily transpiration for each tree, and transparent dashed lines are the cumulated percent of leaf shed for each tree.

After starting the drought experiment at DOY 212, transpiration of all six measured *P. sylvestris* saplings decreased from 1–2.6 kg tree^−1^ day^−1^ to about 10% of the starting value within 2 weeks (DOY 226). Within this period, transpiration was strongly reduced for 2 days (DOY 216–217), most likely because of exceptionally cloudy days with low temperature conditions outside the greenhouse that were not compensated (see Supplementary Figure 2). From DOY 227 onward, tree transpiration almost stopped, with median [95% CI] values of 0.034 [0.001–0.19] kg tree^−1^ day^−1^ (Figure 5). At the same time, leaf shedding increased, which was only marginal during the initial phase of the experiment (0.2 [0.02–0.2] % day^−1^ between DOY 212 and 225). Once transpiration was close to zero, litterfall tripled to 0.55[0.03–0.9] % day^−1^. At the end of the experiment (DOY 265), the trees had lost 30.2% [16.9–37.8] of the initial leaves. We observed that only 2 and 3 year-old needles were shed but never current year needles.

### SOX+ Performance Improved When Including Non-Stomatal Limitations of Photosynthesis

**Figure 6:**
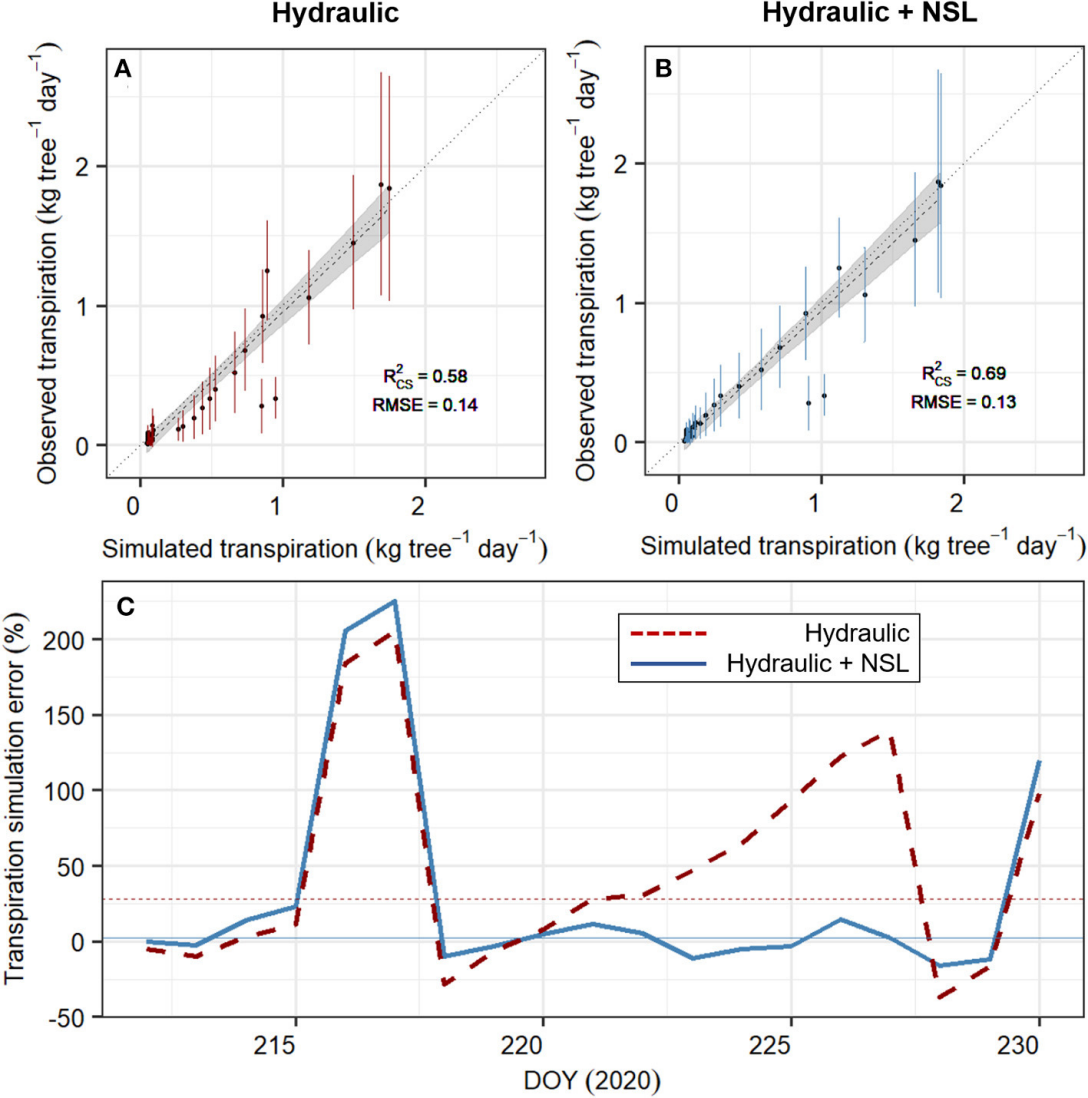
Performance of the SOX+ model calibrated without (“Hydraulic,” **A**, in red) and with (“Hydraulic + NSL,” **B**, in blue) non-stomatal limitations of photosynthesis. **(A,B)** show the comparison between simulated and observed transpiration (in kg tree^−1^day^−1^, points ± SD error bars), during the experiment (DOY 212–265). Daily simulated transpiration is the projected median by sampling 500 times each of the SOX+ model formulation posterior parameter estimates. Noted is the generalized linear model accounting for temporal autocorrelation, model fit ($R_{\text{cs}}^{2}$), and the RMSE. In both formulations, the slope was not significantly different than 1 (*p* < 0.01), and the intercept was not significantly different than 0 (*p* < 0.01). **(C)** represents the SOX+ error in transpiration estimates for the calibration period (DOY 212–230), and for the Hydraulic (red, dashed lines) and Hydraulic + NSL (blue, continuous lines) formulations. Simulation error was calculated as $\frac{Tr_{sim}-Tr_{obs}}{Tr_{obs}}$, where Tr_sim_ is the daily median transpiration from the 500 model, and Tr_obs_ is the daily observed transpiration, both in kg tree^−1^ day^−1^. Horizontal lines represent the median simulated error for each SOX+ formulation.

SOX+ model calibration accounting for non-stomatal limitations (Hydraulic + NSL) outperformed the standard SOX+ model (Figure 6) during the entire experiment. It also provided a more realistic set of posterior parameter estimates, especially regarding *k*_*sr,max*_ (Table 2). For the calibration period (DOY 212–230), the standard “Hydraulic” model setup led to 27.8 ± 17% (Median ± SE) overestimation of daily transpiration, while with “Hydraulic+NSL,” the error reduced to 2.5 ± 16.9% (Figure 6C). Such differences were especially important during the dry-down phase (DOY 220–228). The differences in accuracy between the two model simulations strongly declined during the second half of the experiment, when both model setups resulted in full stomatal closure (gs = g_min_, DOY 240–265).

### NSL and Leaf Shedding Reduce Projected Loss in Root-To-Canopy Hydraulic Conductance

**Figure 7:**
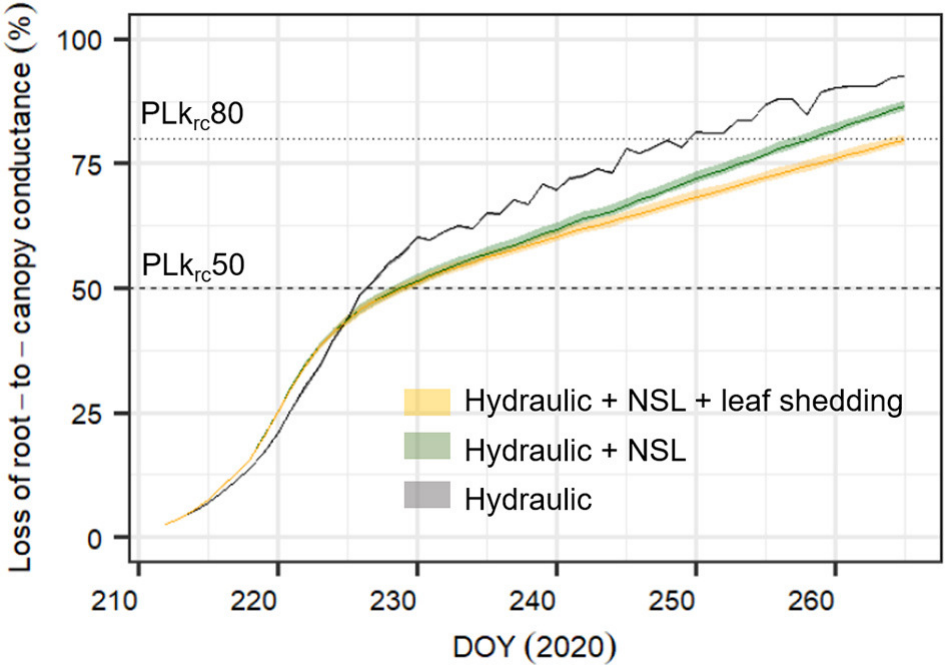
Simulated percent loss in root-to-canopy conductance during the experiment (*PLk*_*rc*_, in %) with three different SOX+ model formulations. Only hydraulic constraints on stomatal conductance (Hydraulic, in gray); hydraulic constrains on stomatal conductance and non-stomatal limitations of photosynthesis accounting for equal leaf area during the whole experiment (Hydraulic + NSL, in green); and hydraulic constraints on stomatal conductance, non-stomatal limitations of photosynthesis, and observed leaf shedding (Hydraulic + NSL + leaf shedding, in orange). Posterior parameter distributions are different for (Hydraulic) and (Hydraulic + NSL, Hydraulic + NSL + leaf shedding), see Table 2. For each model formulation, the figure shows the daily pre-dawn median ± 95% CI *PLk*_*rc*_, obtained from running the simulation by sampling 500 times the posterior parameter distribution. Horizontal lines indicate when *k*_*rc*_ = 0.5 * *k*_*rc,max*_ (*PLk*_*rc*_*50*, dashed line), and when *k*_*rc*_ = 0.2 * *k*_*rc,max*_ (*PLk*_*rc*_*80*, dotted line), a threshold that has been found to be critical for pine survival probability (Hammond et al., 2019).

**Figure 8:**
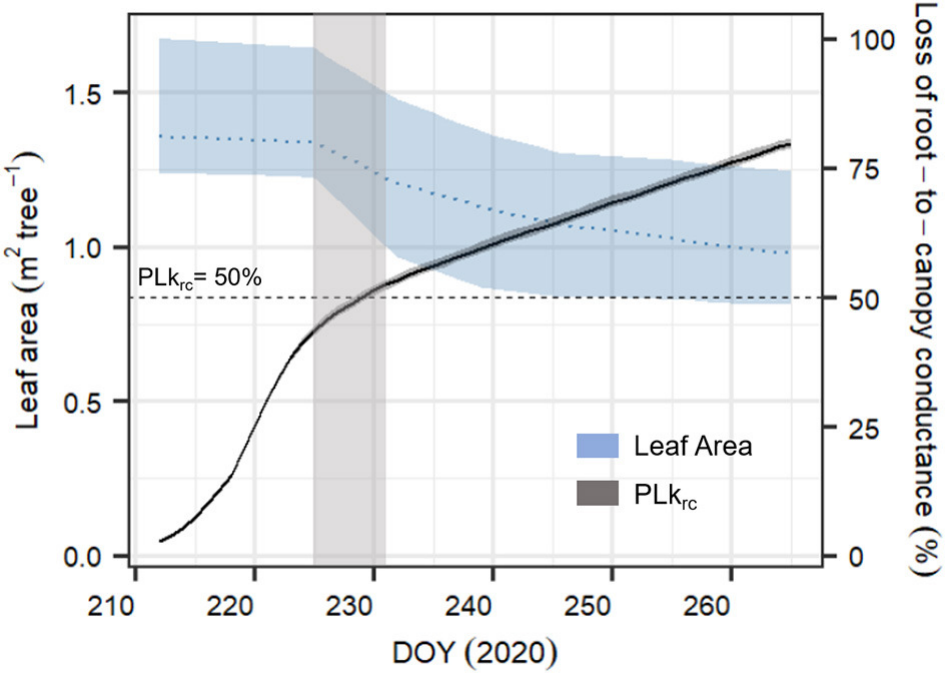
Time series of averaged leaf area (in m^2^ tree^−1^, blue dashed line) for the six monitored *Pinus sylvestris* saplings and simulated dynamics in percent loss in root-to-canopy conductance (*PLk*_*rc*_, in %, black continuous line) according to the complete SOX+ model scheme (Hydraulic + NSL + leaf shedding). Daily leaf area is represented as the mean ± SD. Daily simulated *PLk*_*rc*_ is represented as the median ± 95% CI after 500 model simulation runs by sampling the posterior parameter estimate. The horizontal line indicates when *k*_*rc*_ = 0.5 * *k*_*rc, max*_ (*PLk*_*rc*_*50*, dashed line), while the light gray area represents the uncertainty of when the leaf shedding rate accelerated. Because of weekly sampling campaigns, the area is relatively broad.

**Figure 9:**
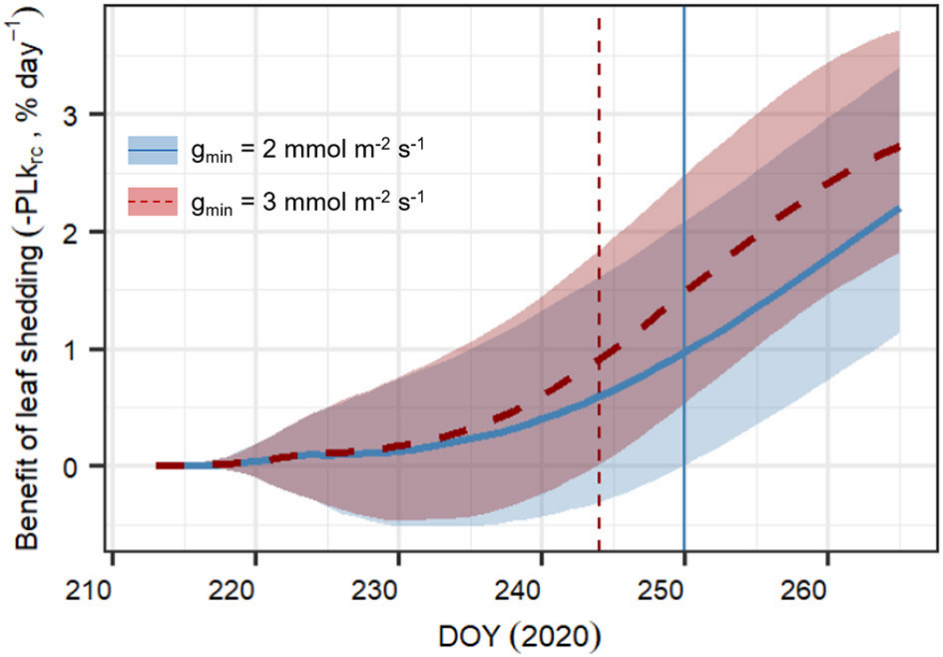
Daily averaged cumulated benefit of observed leaf shedding in preventing loss in root-to-canopy conductance (*PLk*_*rc*_, in %) for minimum leaf conductance (g_min_) = 2 mmol m^−2^ s^−1^ (blue continuous line), and g_min_ = 3 mmol m^−2^ s^−1^ (red dashed line). Results represent the median ± 95% CI of 500 model simulations obtained by randomly sampling the posterior parameter distribution for each of the following scenarios and their combination:with and without leaf shedding, and the two g_min_ values considered. The daily average cumulated difference in *PLk*_*rc*_ between shedding and no shedding is calculated according to Equation 14. The vertical lines for both scenarios indicate the DOY at which the benefit of leaf shedding is significantly >0.

Compared to the original “Hydraulic” SOX+ setup, projected percent loss in root-to-canopy conductance (*PLk*_*rc*_, in %) at the end of the experiment when accounting for NSL (Hydraulic + NSL) was smaller by 6.3 ± 0.2%. Including observed leaf shedding (Hydraulic + NSL + leaf shedding scenario) further reduced projected *PLk*_*rc*_ by a 13 ± 0.2 % (Figure 7). Interestingly, with NSL and leaf shedding considered, the median simulated *PLk*_*rc*_after 54 days of water shortage was below 80%, an important threshold for pine mortality (Hammond et al., 2019). Leaf shedding speed drastically increased between DOYs 225 and 231, when trees had lost between 44 and 53% of their root-to-canopy conductance, according to the SOX+ model (Figure 8) and their stomata were fully closed (daily maximum gs <10% of maximum simulated gs). After full stomatal closure, tree water losses were highly dependent on leaf leakiness, here summarized as g_min_. Increasing g_min_ by 50% resulted in shedding of leaves that was significantly beneficial in reducing *k*_*rc*_ losses compared with no shedding leaves 6 days earlier than assuming g_min_ = 2 mmol m^−2^ s^−1^ (DOY 244 vs. DOY 250). The benefits of leaf shedding were significantly higher at g_min_ = 3 mmol m^−2^ s^−1^ than at g_min_ = 2 mmol m^−2^ s^−1^ after DOY 232 (*p* < 0.05, after a Kolmogorov–Smirnov non-parametric test), and for the rest of the period simulated (Figure 9).

## Discussion

The findings suggest that non-stomatal limitations (NSL) of photosynthesis and leaf shedding processes play an important role as hydraulic safety mechanisms in *P. sylvestris*, as they reduce losses in root-to-canopy conductance during severe drought stress (Figure 7). Drought-induced leaf shedding in *P. sylvestris* saplings started when predicted loss of root-to-canopy hydraulic conductance was close to 50%. This is in accordance with the “hydraulic fuse” hypothesis, which states that cavitation in leaves would occur earlier than xylem cavitation to avoid irreversible xylem conductance losses (e.g., Hochberg et al., 2017, Choat et al., 2018), as leaves are cheaper to rebuild than new vessels in the xylem (Tyree and Ewers, 1991). The results also concur with previous observations of leaf shedding delaying the time to reach lethal dehydration thresholds (Blackman et al., 2019) while also identifying g_min_ as a key trait involved in plant dehydration processes (Duursma et al., 2019; Cochard, 2020). These findings highlight the importance of including non-stomatal processes (i.e., NSL and leaf area adjustments) into simulation models in order to account for tree physiological feedback responses to drought stress.

### Non-Stomatal Limitations of Photosynthesis as a Hydraulic Safety Mechanism

Including NSL to restrict photosynthesis improved the SOX+ model representation of observed transpiration reductions during the dehydration phase, especially when stomata were not fully closed. Model improvements based on similar observations have been reported previously (e.g., Yang et al., 2019; Gourlez de la Motte et al., 2020). Including NSL reduced projected losses in root-to-canopy conductance by overall 6% after 54 days without irrigation. It also delayed reaching 80% of *PLk*_*rc*_, an important threshold for pine mortality (Hammond et al., 2019), by 8 days. As plants dehydrate, considering NSL led to lower optimum stomatal conductance per photosynthetic gain (i.e., it led to decreased δ*An*/δ*C*_*i*_ in Equation 8). We identified a Ψ_*canopy*_ reference value of −1.7 MPa for a 50% strength of the NSL limitation, this value being lower than the observed Ψ_*xylem*, 50_ (−2.6 MPa). This implies that NSL mechanisms are acting at the onset of embolism formation, which may be indicative of an active hydraulic safety mechanism (Choat et al., 2018).

We acknowledge that simulating NSL as a decline in apparent V_*cmax*_ and J_*max*_ summarizes the effects from a multiplicity of interacting plant physiological responses to drought, which might be better considered separately. For example, water and carbon transport in the mesophyll might be described as a function of aquaporin expression in dependence on cell water potential (Flexas et al., 2012; Paudel et al., 2019), and the effects on photosynthesis could be separated into suppression of rubisco regeneration (Rizhsky et al., 2002; Pilon et al., 2018), and reduction in rubisco activity. Also, reduced sink strength of tissue experiencing drought limitations (Hsiao et al., 1976; Fatichi et al., 2014) could eventually be used to describe the down-regulation of photosynthesis explicitly, e.g., *via* higher leaf sugar concentration due to reduced phloem load (Riesmeier et al., 1994; Sevanto, 2014). These mechanisms, however, have not been sufficiently resolved to be used in general models.

### Shedding Leaves After Stomatal Closure Buffered Projected Loss in Conductance

Reducing leaf area is a well-known phenomenon that decreases whole tree transpiration (e.g., Whitehead et al., 1984; Martínez-Vilalta et al., 2014; Wolfe et al., 2016). During the first phase of the dry-down, leaf shedding was only marginal, but it increased after full stomatal closure (Figures 5, 8), when reducing leaf area was the only possibility to further reduce leaf leakiness and leaf C maintenance cost. We found that leaf shedding reduced projected *k*_*rs*_ losses by 7% at the end of the experiment. Also, when including leaf shedding dynamics, the pines did not reach the lethal *PLk*_*rc*_*80* threshold. Interestingly, increasing g_min_ by 50% further underscored the benefits of leaf shedding, which then even started earlier during the dry-down. Since g_min_ seems to depend on environmental conditions during leaf development (Duursma et al., 2019) and also increases with leaf temperature (e.g., Schuster et al., 2016; Cochard, 2020), leaf shedding may represent an acclimation strategy to counteract leaf leakiness during later growth stages (Blackman et al., 2019) and heat waves. This is supported by findings that less sclerophyllous trees (i.e., those with higher g_min_) are likely to shed more leaves earlier during heat-drought stress development (e.g., Ogaya and Penuelas, 2004; Montserrat-Marti et al., 2009).

In accordance with recent findings (Wolfe et al., 2016; Cardoso et al., 2020; Li et al., 2020), Scots pine shed its 2 and 3 year-old needles once k_rs_ was halved and leaf conductance was strongly reduced (approximately gs = g_min_). This implies that in *P. sylvestris*, leaf shedding has likely been triggered as a last-chance hydraulic safety mechanism, as pines do not re-grow shed leaves until the next growing season. This may lead to severe C uptake reductions after drought release, especially if the drought occurs early during the growing season (Eilmann et al., 2013), with consequences that may extend up to 4 years after drought (e.g., Galiano et al., 2011). In the experiment, the trees shed about 30% of their leaves, which may imply an equivalent loss in photosynthesis capacity after re-watering. Such C uptake reductions may still have severe implications for post-drought xylem and canopy recovery (e.g., Yan et al., 2017; Ruehr et al., 2019; Kannenberg et al., 2020). However, negative impacts of leaf losses are probably less severe because mostly the older leaves, which are less efficient in terms of photosynthesis, were shed first (Escudero and Mediavilla, 2003; Niinemets et al., 2005, but see Poyatos et al., 2013). Also, the C balance during drought is less negative because of reduced maintenance respiration; thus, lower depletion of non-structural carbohydrates reserves is expected. The inclusion of the SOX+ model in a whole-tree dynamics simulation model may shed more light upon the benefits and drawbacks of leaf shedding in terms of whole tree C balance and growth.

### Uncertainties and Lines to Proceed Further

Trees may respond to soil drought with various physiological reactions in order to save water and protect their conductive tissue. Besides stomatal regulation of evaporation demand and potential photosynthesis, additional processes are involved that affect the relationship between stomatal opening and photosynthesis activity, particularly under drought as has been described, e.g., by Eller et al. (2018, 2020). In this study, we have accounted for these processes in a lumped way by training a process-based tree hydraulic model (SOX+) from “*in situ*” observations (e.g., Medlyn et al., 2015; Dietze et al., 2018). However, we acknowledge that the experimental basis for this new mechanism is still poor, based on a small number of repetitions and a relatively high variability in individual plants. Also, it would be more convincing that loss in xylem conductance is a driving force for drought responses if it would have been directly measured instead of indirectly calculated from water potential and transpiration. It should also be noted that the additional parameters required to describe the new model features are yet uncertain with respect to their generality; thus, further studies are needed to evaluate their precision, species-dependency, or relationship to wood or leaf anatomical traits (Schumann et al., 2019, Velikova et al., 2020).

Similar caution needs to be applied to the second investigated process of leaf shedding. It seems that there is a clear threshold of loss in root-to-canopy hydraulic conductance at which leaf shedding began. Therefore, we suggest a generalization of drought-induced leaf shedding dynamics that may be included in simulation models accounting for tree hydraulics. This is based on a basal leaf turnover rate and a drought-induced leaf shedding rate that is considered after a given threshold of loss in hydraulic conductanceis reached:

(15a) $$\begin{array}{l} LA_{i}=\;LA_{\left(i-1\right)}-basal_{LS}LA_{\left(i-1\right)}ifPLk_{rc}<x\; \end{array}$$

(15b) $$\begin{array}{l} LA_{i}=\;LA_{\left(i-1\right)}-drougth_{LS}LA_{\left(i-1\right)}ifPLk_{rc}\geq x\; \end{array}$$

where LA_i_ is the whole tree leaf area on day “i” (in m^2^ tree^−1^), basal_LS_ is the basal rate of leaf shedding without drought stress, drought_LS_ is the leaf shedding rate under drought stress, and x is the threshold of conductance loss at which drought-induced leaf shedding occurs. For instance, in the experiment (Section Leaf Shedding Speed Increased Concurrently With Transpiration Stop), basal_LS_ would equal to 0.002, drought_LS_ to 0.0055, and x to 50%. According to recent observations, 50% loss in hydraulic conductance seems to be a realistic threshold, occurring within a wide range of environments and plant types (e.g., Wolfe et al., 2016; Cardoso et al., 2020; Li et al., 2020). Nonetheless, we acknowledge that depending on the species-specific strategies to face hydraulic stress, such threshold may vary (e.g., Ruehr et al., 2016). We hypothesize further that this strategy may be linked to differences in g_min_, as the benefits of leaf shedding appear earlier and to a larger degree when leaf leakiness is higher.

The suggested model modifications account for non-stomatal acclimation mechanisms affecting canopy conductance, which are commonly accepted as an important driver of plant water use regulation but are still poorly tested in simulation models. Nevertheless, we are aware that other potential acclimation processes such as changes in rooting depth, root distribution, or soil-to-root conductance (e.g., Mu et al., 2021), as well as changes in leaf distribution or traits, such as leaf thickness and stomatal density, affect whole plant conductance. Also, short-term responses, such as an increase in leaf cuticular conductance (g_min_) in response to rising temperature (e.g., Cochard et al., 2021), are not addressed in this version of SOX+, which leaves room for model improvement. Furthermore, the SOX+ model has, so far, been tested only for small trees growing under controlled conditions. Thus, model performance should be assessed under field conditions, particularly for trees of different sizes and species. Still, the inclusion of NSL and leaf shedding processes enhanced model transpiration estimates, which were identified as key mechanisms that trees trigger to buffer conductance losses under drought stress.

### Relevance for Climate-Change Scenario Analyses

Including the proposed processes into ecosystem models would improve water consumption estimates during drought and hot drought events. Because heat waves alongside increased VPD are supposed to increase in intensity and frequency (Basarin et al., 2020), this may potentially improve model projections of forest drought responses. Parametrizing species in order to consider tradeoffs between elongating carbon gain and reducing hydraulic stress *via* leaf shedding will also enable to better represent the differences in competition strength between tree species under future environmental conditions. This is particularly useful in ecosystem models addressing the interactive impacts of increased atmospheric CO_2_ and a hotter and drier climate on forests. In addition, the mechanistic simulation of loss in xylem conductance also considering feedback responses may lead to an improvement in tree mortality process description (Bugmann et al., 2019). In contrast to empirical approaches, it allows to consider species- and environmental-specific adaptation strategies, represented by species traits that indicate different hydraulic vulnerabilities. Since tree mortality is an underrepresented but highly important process when addressing forest dynamics under global warming (Meir et al., 2015), incorporating leaf area acclimation to drought and non-stomatal limitations of photosynthesis in larger-scale forest simulation models will likely improve climate change scenario assessments.

## Data Availability Statement

The datasets presented in this study can be found in online repositories. The names of the repository/repositories and accession number(s) can be found at: Code and data available at: https://zenodo.org/badge/latestdoi/381126647.

## Author Contributions

DN-S wrote the initial version of the manuscript, implemented SOX+ model formulation, and analyzed the data. DN-S, NR, and BB designed and implemented the experiment. RG and NR assisted with manuscript writing and research design and supervised the data analysis. TK analyzed the xylem vulnerability curves. BB, TK, RR, and SS collaborated for the manuscript content and collected field data. All the authors contributed to manuscript editing and writing.

## Conflict of Interest

The authors declare that the research was conducted in the absence of any commercial or financial relationships that could be construed as a potential conflict of interest.

## Publisher's Note

All claims expressed in this article are solely those of the authors and do not necessarily represent those of their affiliated organizations, or those of the publisher, the editors and the reviewers. Any product that may be evaluated in this article, or claim that may be made by its manufacturer, is not guaranteed or endorsed by the publisher.
