# Supplementary material for: Leaf Shedding and Non-Stomatal Limitations of Photosynthesis Mitigate Hydraulic Conductance Losses in Scots Pine Saplings During Severe Drought Stress
Source: Front Plant Sci. 2021 Sep 3;12:715127. doi: 10.3389/fpls.2021.715127 (PMC8448192; doi:10.3389/fpls.2021.715127)
Supplement: Supplementary file 1 [file Data_Sheet_1.docx]

Leaf shedding and non-stomatal limitations of photosynthesis mitigate hydraulic conductance losses in Scots pine saplings during severe drought stress

Daniel Nadal-Sala, Rüdiger Grote, Benjamin Birami, Timo Knüver, Romy Rehschuh, Selina Schwarz, Nadine K. Ruehr

**Supplementary material**

**Tables:**

| **Table S1.** Comparison of the different biomass compartments weight between the monitored trees and the non-monitored trees. Leaf, root and wood biomass are provided in g dry weight, and specific leaf area (SLA) is provided in cm^-2^ g^-1^ leaf. All data are provided as mean [95%CI]. N is the number of the individuals for each group. As the data did not follow a normal distribution for any of the variables, significant differences were assessed with a Kolmogorov-Smirnov non-parametric test. Different letters indicate statistically significantly differences between groups with p<0.05. | | | | | |
| --- | --- | --- | --- | --- | --- |
| Trees | Leaf (g dry weight) | Root (g dry weight) | Wood (g dry weight) | SLA (cm^2^ g^-1^) |  |
| Monitored | 323.4 [292.3-386.4]^a^ | 184.5 [131.0-217.6]^a^ | 401.8 [336.0-470.1]^a^ | 42.3 [34.3-46.8]^a^ | 6 |
| Non-monitored | 333.8 [221.6-467.0]^a^ | 145.5[ 85.5-195.2]^a^ | 354.0 [213.8-457.2]^a^ | 42.2 [37.2-45.6]^a^ | 10 |

**Figures:**


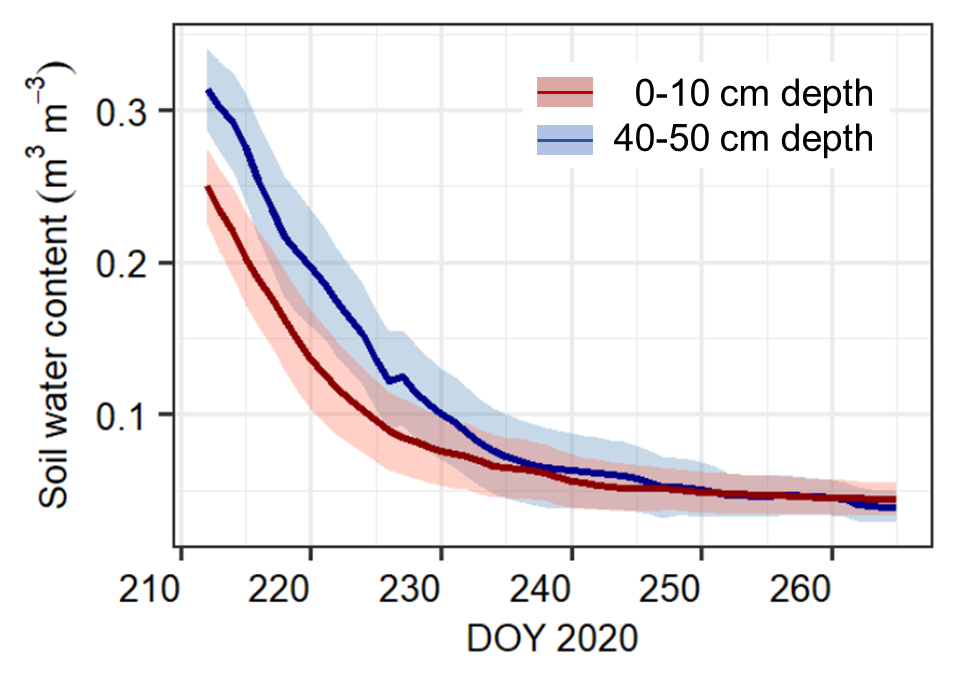


**Figure S1.** Soil water content (SWC, in m^3^ m^-3^) evolution along the dry-down experiment for potted *Pinus sylvestris* saplings in the glasshouse of the IMK-IFU (n = 6), at two different measuring depths. Represented are pre-dawn daily SWC averages ± 1SD.


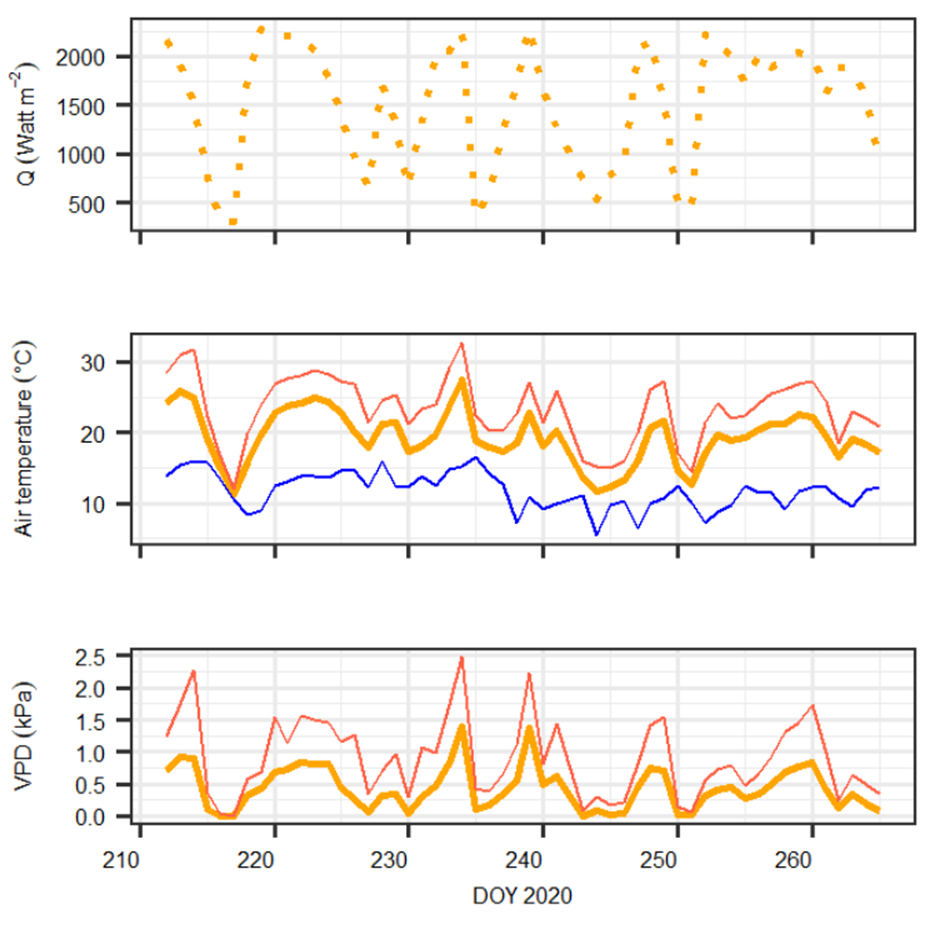


**Figure S2.** Daily meteorological conditions recorded by the IMK-IFU meteorological station during the experiment. Upper panel: daylight average incoming radiation (Q, in Watt m^-2^); middle panel: maximum (red), minimum (blue) and average (orange) air temperature (in °C); bottom panel: maximum (red), and average (orange) atmospheric vapor pressure deficit (VPD, in kPa). Minimum daily VPD is not represented because it was 0 or lower each night. Noteworthy are the extremely low Q, temperature and VPD values during DOYs 216-217.
